# Viscoelastic Hemostatic Testing as a Diagnostic Tool for Hypercoagulability in Liver Transplantation: A Narrative Review

**DOI:** 10.3390/jcm13206279

**Published:** 2024-10-21

**Authors:** Khaled Ahmed Yassen, Dur I Shahwar, Aqeel Qasem Alrasasi, Feras Aldandan, Danah Sami Alali, Maryam Yousef Almuslem, Nouran Hassanein, Imtiyaz Khan, Klaus Görlinger

**Affiliations:** 1 Anaesthesia Unit, Surgery Department, College of Medicine, King Faisal University, P.O. Box 400, AlAhsa 31982, Saudi Arabia; shahwardr@gmail.com; 2 Alumini, College of Medicine, King Faisal University, P.O. Box 400, AlAhsa 31982, Saudi Arabia; a_q_rasasi@hotmail.com (A.Q.A.); danasalali9@gmail.com (D.S.A.); meme.yousef@hotmail.com (M.Y.A.); 3 Alumini, College of Medicine, Alfaisal University, P.O. Box 50927, Riyadh 11533, Saudi Arabia; nhassanein@alfaisal.com; 4 Surgery Department, College of Medicine, King Faisal University, P.O. Box 400, AlAhsa 31982, Saudi Arabia; ahmad123c@gmail.com; 5 Medical Director, Tem Innovations GmbH, 81829 Munich, Germany; kgoerlinger@werfen.com

**Keywords:** liver, hypercoagulability, thromboelastometry, thromboelastography, liver transplantation

## Abstract

Liver transplantation is a complex surgical procedure in which various forms of coagulation dysfunction can occur, including perioperative hypercoagulability. The hemostasis balance in liver graft recipients with end-stage liver disease can shift to thrombosis or haemorrhage, depending on the associated risk factors and clinical conditions. Hypercoagulability can result in serious complications such as thromboembolism, which can affect the vessels of the newly transplanted liver graft. Standard coagulation tests (SCTs), such as prothrombin time and activated partial thromboplastin time (aPTT), have a poor ability to diagnose and monitor an early stage of hypercoagulability. Recent studies demonstrated that viscoelastic hemostatic elastic tests (VETs), such as rotational thromboelastometry (ROTEM) and thromboelastography (TEG), are promising alternative tools for diagnosing hypercoagulability disorders. VETs measure clotting and clot formation time, clot strength (maximum clot firmness), fibrin and platelet contribution to clot firmness, and fibrinolysis, which makes them more sensitive in identifying liver graft recipients at risk for thrombosis as compared with SCTs. However, developing evidence-based guidelines for the prophylaxis and treatment of hypercoagulability based on VET results is still needed.

## 1. Introduction

Patients with end-stage liver disease (ESLD) frequently show a rebalance of hemostasis. However, this volatile rebalance can quickly switch to bleeding or thrombosis. Hemostasis depends on the equilibrium between procoagulants and anticoagulants as well as fibrinolytic and antifibrinolytic factors. Hypercoagulability or thrombophilia may result in thromboembolic complications, such as portal vein thrombosis or hepatic artery thrombosis, and increase the perioperative morbidity and mortality in liver transplant recipients [1,2].

Standard laboratory coagulation tests (SCTs) are unable to discriminate between hypo- and hypercoagulability and, therefore, may result in overtreatment. This can result in inappropriate blood transfusion, transfusion-associated circulatory overload (TACO), and portal hypertension, which again may aggravate bleeding. Viscoelastic hemostatic testing (VET), such as thromboelastometry (ROTEM) and thrombelastography (TEG), often demonstrates normal coagulation or even hypercoagulability in contrast to SCT [3,4,5].

The perioperative challenge faced by anesthesiologists is that SCTs have a poor ability to detect hypercoagulability. Furthermore, SCTs (prothrombin time (PT), activated partial thromboplastin time (aPTT), and platelet count) are of questionable value in the perioperative setting due to their long turnaround time and their inability to adequately reflect the immediate complex changes in hemostasis. These VET devices can diagnose these changes in hemostasis in real-time and at the bedside (point of care), but they are not available in every liver transplant center and require education and training [6,7,8]. Recent versions of VET devices are easier to operate and more user-friendly due to the automatization of measurements. In contrast to SCTs, VETs monitor blood clot elasticity and both the cellular and plasmatic blood components of the clotting process.

## 2. Aim and Method of the Narrative Review

This review aims to explore the published literature that identifies and describes both intra- and postoperative hypercoagulability findings as demonstrated by VETs in adult liver transplant recipients

Medline, Scopus, PubMed, and Google Scholar were used to search for the English-published literature between 15 January 1995 and 15 May 2024. Current peer-reviewed and accepted online ahead-of-print publications were also included. PubMed was searched using the following mesh-created keywords: Liver; Hypercoagulation; Thromboelastometry; Thromboelastography; and Liver Transplantation. Keywords were selected from the National Library of Medicine, Medical Subject Headings database (https://www.nlm.nih.gov/mesh/MBrowser.html, accessed on 7 October 2024).

The studies retrieved included the following: randomized controlled trials (RCTs), interventional studies, observational studies, prospective studies, retrospective studies, case series, and reports, as well as reviews that describe hypercoagulability during and after liver transplantation with VET devices, the site of thrombosis, and the outcome of the recipients with thromboembolic events. The results of VETs were compared to SCT findings.

Fourteen publications presented in Table 1 [1,7,8,9,10,11,12,13,14,15,16,17,18,19] simultaneously describe the coagulation findings by VETs compared to SCTs.

## 3. Rotational Thromboelastometry

ROTEM, Tem Innovations, Munich, Germany, is a point-of-care device that is capable of evaluating the viscoelastic properties and kinetics of the whole coagulation process for both extrinsic and intrinsic pathways, including clot formation and lysis, in vitro [9,20]. ROTEM measures the viscoelastic characteristics of a blood clot and the interactions between various coagulation-promoting factors and inhibitors. The five principal tests used with ROTEM include EXTEM, INTEM, FIBTEM, APTEM, and HEPTEM, which assess the extrinsic coagulation pathway (EXTEM), intrinsic coagulation pathway (INTEM), fibrin contribution to clot firmness (FIBTEM), effect of antifibrinolytic drugs (APTEM), and the effect of heparinase on intrinsically activated blood samples (HEPTEM), respectively.

EXTEM measures tissue factor activation and provides information about the extrinsic coagulation pathway, similar to PT/INR. INTEM measures contact activation and provides information about the intrinsic coagulation pathway, similar to aPTT. FIBTEM uses cytochalasin D, an actin polymerization inhibitor, to block platelet contribution to clot formation, leaving only the impact of fibrin formation and polymerization to be measured.

**Figure 1:**
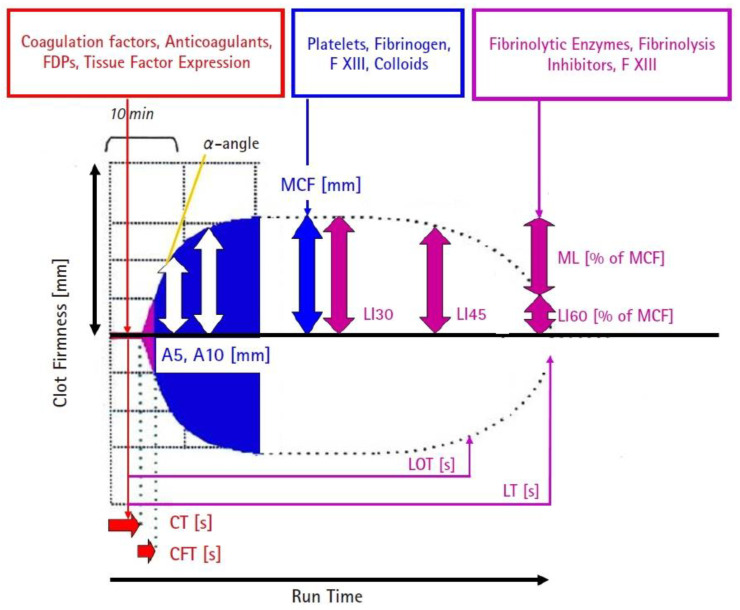
ROTEM parameters and indices. CT: coagulation time in seconds (time from test start to 2 mm clot firmness). CFT: clot formation time in seconds (time from 2 to 20 mm clot firmness). A5: clot firmness amplitude in 5 min. A10: clot firmness amplitude in 10 min. FDPs: fibrinogen degradation products. F XIII: coagulation factor XIII. LI30: lysis index 30 min after CT in % (residual clot firmness in percentage of MCF). LI45: lysis index 45 min after CT in %. LI60: lysis index 60 min after CT in %. LOT: lysis onset time in seconds (time from CT to 15% fibrinolysis = 85% residual clot firmness compared to MCF). LT: lysis time in seconds (time from CT to 90% fibrinolysis = 10% residual clot firmness compared to MCF). Courtesy of Klaus Görlinger, Munich, Germany [3].

ROTEM measures the following parameters: coagulation time (CT), clot formation time (CFT), A5 (clot firmness amplitude 5 min after CT), A10 (clot firmness amplitude 10 min after CT), and maximum clot firmness (MCF). CT is the time needed to initiate clot initiation (until clot firmness reaches 2 mm). CFT characterizes clot kinetics based on the time needed to increase clot firmness from 2 to 20 mm. MCF is the maximum clot firmness measured in mm achieved during the thromboelastometric measurement (Figure 1). Several studies demonstrated that an EXTEM or INTEM MCF of >68 mm (A10 > 61.5 mm) is a strong indicator of hypercoagulability and a predictor of thrombosis. Furthermore, a FIBTEM MCF > 25 mm is associated with a five-fold increased risk of portal vein thrombosis [6,21,22].

## 4. Thromboelastometry (TEG)

**Figure 2:**
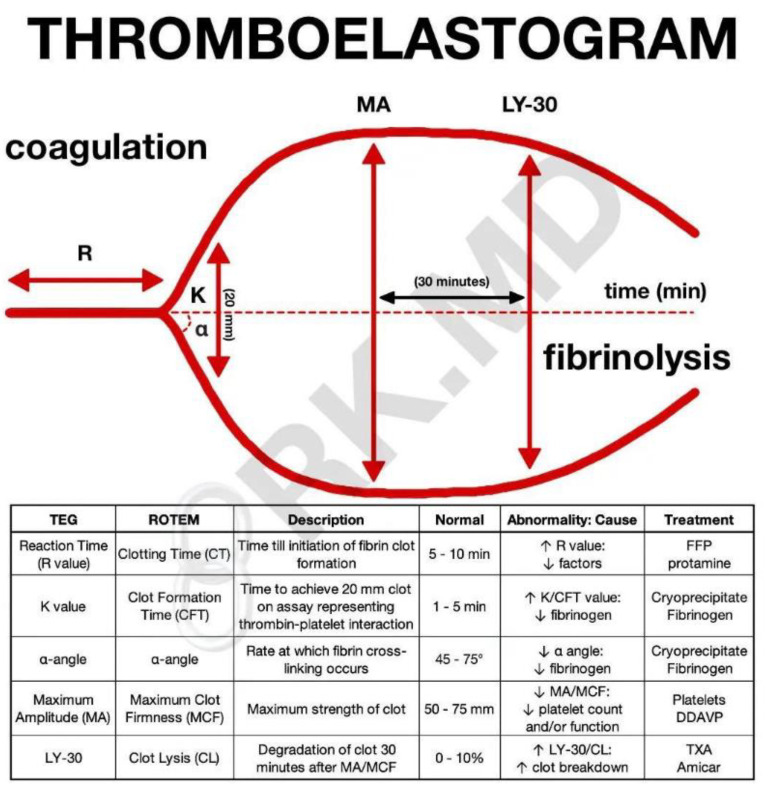
Thromboelastography parameters and indices. R (reaction time): time elapsed till clot initially forms. K (K time): time elapsed till the clot reaches a fixed strength of 20 mm. Angle (clot kinetics): speed of fibrin accumulation. MA (maximum amplitude): highest vertical amplitude. LY30 (lysis 30 min after MA): percentage of amplitude reduction 30 min after MA. Courtesy of Rishi Kumar, MD, USA. https://rk.md/2014/thromboelastography-teg/ (accessed on 3 January 2024); https://images.app.goo.gl/nQmbuBxBahWeEuAT7 (accessed on 3 January 2024).

The standard kaolin-activated TEG was used to assess maximal clot strength and detect hypercoagulability in citrated blood samples. TEG (Haemonetics Corp, Boston, MA, USA) parameters are the following: the R-time (3–8 min), which is the reaction time characterizing clot initiation; the K-time (1–3 min) describes the kinetics of clot formation; and maximum amplitude (MA, 51–69 mm) reflects the maximum clot firmness amplitude based on fibrin–platelet interactions. Any MA result > 69 mm was defined as hypercoagulability; Figure 2 [23,24].

Regarding the measurement principle, the cup is slowly oscillating in TEG and changes in viscoelasticity are measured by a suspended pin attached to a torsion wire. In contrast, the cup is fixed in ROTEM while the pin is oscillating and changes in viscoelasticity are detected by a LED–mirror–light detector system. Furthermore, the pin is stabilized by a ball-bearing, which allows for its mobile use at the bedside of the patient. In addition, ROTEM enables a precise discrimination between fibrin and platelet contribution to clot firmness by combining the assays EXTEM and FIBTEM [25].

**Standard coagulation tests (SCTs):** SCTs include the activated partial thromboplastin time (aPTT, 30–40 s), prothrombin time (PT, 11 to 13.5 s), international normalized ratio (INR, 0.8–1.1), and the plasma fibrinogen concentration (Clauss method, 2 to 4 g/L). SCTs are usually measured semi-automatically in citrated platelet-poor plasma. Therefore, the contribution of the platelet to the coagulation process is missing. Platelet count is usually measured in ethylenediamine tetraacetic acid (EDTA) with a Coulter Counter [26,27,28,29,30]. EDTA is a chelating agent used to treat heavy metal toxicity. EDTA does not distort red blood cells, but it can affect platelet count when measured by automated hematological analyzers (pseudothrombocytopenia).

Hypercoagulability, as defined by SCTs, is discussed controversially. Here, an abnormally short aPTT and PT, and low aPTT and PTratios or INR are considered, by some clinicians, as signs of hypercoagulability. This includes an aPTT ratio of ≤0.9 as defined by Legnani et al. in 2006 and Senthil et al. in 2014 [31,32]. However, a detailed hypercoagulability workup should include several factors such as fibrinogen, D-dimer, factor VIII, protein C and S, antithrombin III, prothrombin gene mutation, factor V Leiden, Lupus Anticoagulants, and glycoprotein antibodies. In contrast to VET, these tests are not for continuous monitoring during surgery and the results are not immediately available [33,34].

## 5. Discussion

### 5.1. Rebalanced Hemostasis in Liver Patients

**Figure 3:**
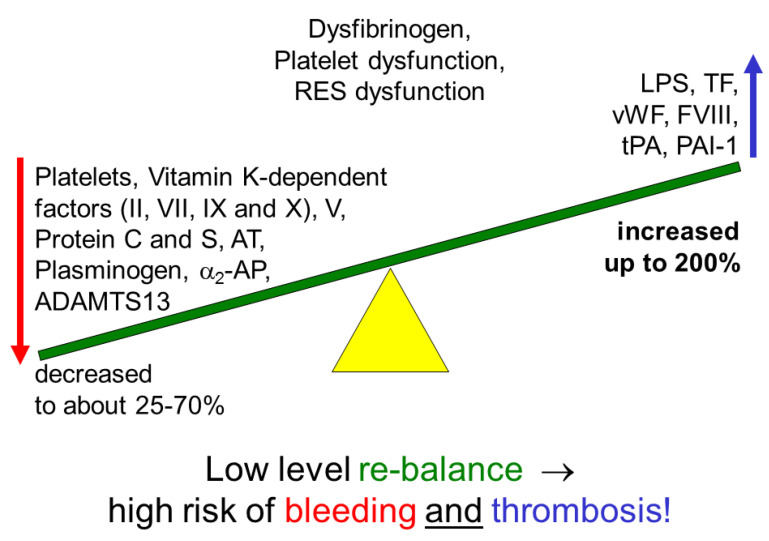
Hemostasis in hepatic patients with cirrhosis is rebalanced at a low level associated with a high risk of bleeding and thrombosis. Courtesy of Klaus Görlinger, Essen, Germany.

Normal hemostasis requires a delicate balance between pro- and anticoagulants and an equilibrium between fibrinolysis and fibrinolysis inhibitors [3,4,35,36]. If this balance is not maintained, hypocoagulability or hypercoagulability will prevail. The hepatocytes in ESLD can affect this balance by not producing enough vitamin K-dependent coagulation factors (II, VII, IX, and X) or vitamin K-dependent inhibitors such as protein C and S. Antithrombin and factor V production is also reduced (Figure 3). In contrast, the non-hepatic vascular endothelium produces and releases more von Willebrand factor (vWF) and coagulation factor VIII. This is even reinforced by a significant reduction in the activity of the vWF-cleaving enzyme ADAMTS13 produced in the liver, which can lead to the formation of microthrombi [37,38,39,40]. In ESLD, the plasminogen and alpha2-antiplasmin levels decrease, while tissue plasminogen activator (tPA) and plasminogen activator inhibitor-1 (PAI-1) levels increase [41]. Infection and sepsis can induce disseminated intravascular coagulation (DIC). Recent studies showed evidence for a shutdown of fibrinolysis rather than hyperfibrinolysis in acute liver failure. Recipients with liver cirrhosis are more prone to thrombosis than bleeding, despite signs of hypocoagulability in SCTs [41,42,43,44].

### 5.2. Can VET Diagnose Hypercoagulability During and After Liver Transplantation?

**Figure 4:**
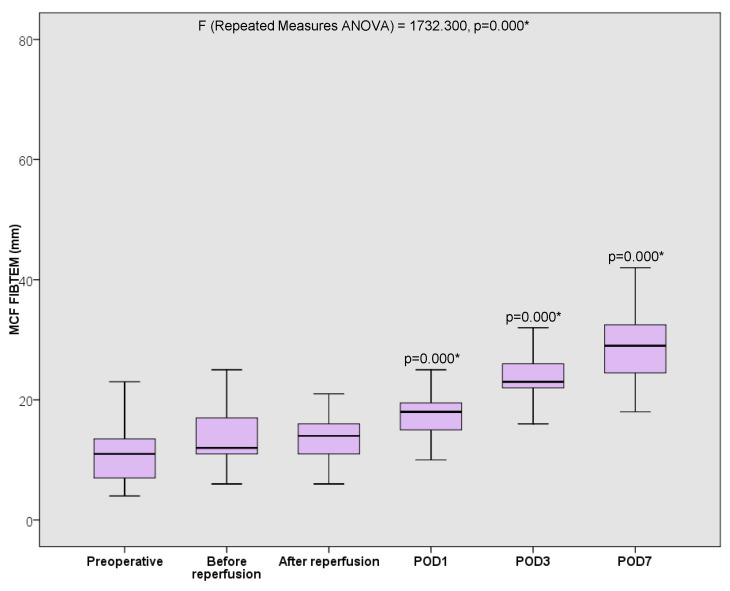
Box–whisker plots for perioperative changes in FIBTEM MCF (reference range 9–25 mm) for the 50 liver transplant recipients. Results are expressed as maximum, minimum, median (line within the box), and 25th and 75th percentiles (error bars) at the selected time points. Repeated measures of ANOVA were used; FIBTEM MCF changes were significant all over the measuring point, * *p* < 0.001. P values above each box indicate their significance when compared to the pre-operative value using a pair-wise comparison with Bonferroni adjustment for multiple comparisons (*p* < 0.01 is considered statistically significant). POD = postoperative day. Courtesy of Yasmin Kamel, Menoufia University, Egypt [7].

**Figure 5:**
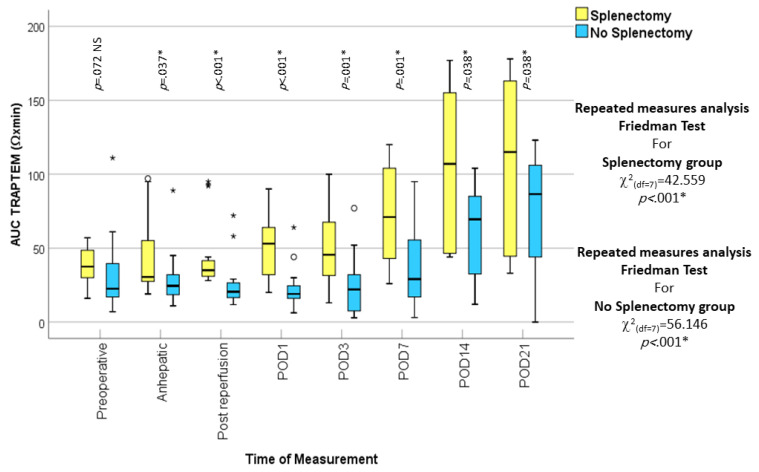
Box and whisker graph of AUC TRAPTEM (Ω × min; RR, 55–154 Ω × min). The thick line in the middle of the box represents the median; the box represents the inter-quartile range (from 25th to 75th percentiles); the whiskers represent the minimum and maximum after excluding outliers (circles) and extremes (asterisks). The asterisks (*) associated with the _p-value indicates statistical significance. AUC, the area under the aggregation curve; POC, postoperative day. TRAPTEM is a ROTEM delta platelet function parameter that represents the platelet thrombin receptor activating Peptide-6. The figure demonstrates the enhancement of platelet activity with splenectomy during liver transplantation. Courtesy of Eman Hegazy, Menoufia University, Egypt [19].

A few studies have been done to investigate hypercoagulability, as demonstrated by VETs among liver transplant recipients. Kamel et al. published one of those few prospective observational studies, providing evidence of a significant postoperative stepwise increase in the FIBTEM (MCF) associated with an increased risk of thrombosis, particularly on postoperative day 5 and 7. FIBTEM is a ROTEM assay representing fibrin contribution to clot firmness as described in the above Figure 4. This increase in fibrin contribution to clot firmness was not associated with elevated fibrinogen plasma levels [7]. Yassen et al. published another prospective observational study that looked at the perioperative platelet activity measured by ROTEM *platelet* (whole blood impedance aggregometry; Tem Innovations, Munich, Germany) in liver transplant recipients. This study reported a significant increase in platelet activity seven days postliver transplant [18]. Hegazy et al. also assessed the effect of splenectomy during liver transplantation on platelet count and function. They found that splenectomy does enhance the recovery of platelet function as early as postoperative day 3, and that platelet function could exceed the normal range by postoperative day 14–21, as demonstrated in Figure 5 in their publication [19].

The incidence of hypercoagulability in liver transplant recipients can range between 16% and 86% as reported by Kamel et al. in 2018 [7]. They also stated that an increase in FIBTEM MCF (>25 mm) can predict thromboembolic complications in hepatocellular carcinoma and in patients with pre-existing thrombophilic factors (high lupus anticoagulant, and/or positive Factor V Leiden mutation). Krzanicki et al. demonstrated significant intra-operative thromboelastographic (TEG) evidence of hypercoagulability during liver transplantation [8]. In patients with non-hepatic diseases, several ROTEM/TEG studies demonstrated their ability to identify hypercoagulation (EXTEM MCF > 68 mm) and to predict thrombosis in non-cardiac and non-hepatic surgery, in conditions such as sepsis, experimental studies, and mechanical cardiac assist, when SCTs failed to predict a thromboembolic event [21,22,45,46,47,48,49,50].

### 5.3. The Incidence of Thromboembolic Events

Table 1 presents the details of published studies with hypercoagulability in liver transplant recipients and the incidence of thromboembolic events in each study. Despite prolonged conventional coagulation tests, liver transplant recipients have shown normal or hypercoagulable status on several occasions during and after surgery as reported by Lisman et al. in 2010 [44]. This hypercoagulable state can lead to serious thromboembolic events as demonstrated by Arshad et al. in 2013 [51]. The incidence of venous thromboembolism, as reported by Salami et al. in a retrospective review, can reach 4.5% in liver transplant recipients [5]. Individual genetic pre-disposition for hypercoagulability plays an important role in the initiation of thromboembolic events [52,53]. The assessment and treatment of hypercoagulability can benefit from the use of VET devices as stated by Zamper et al. in 2017 [54]. Earlier in 2005, Lerner et al. reported about four liver transplant recipients with intra-operative cardiopulmonary thromboembolism during surgery at their center. All four patients were hypocoagulable on SCTs, but unfortunately, were not monitored with VET due to the lack of VET devices in this center. Two recipients died and two survived. Lerner et al. carried out a systematic review of similar case reports. They were able to trace 13 published intra-operative events. The most significant finding was the ability of VETs to diagnose hypercoagulability when SCTs failed [2]. Later in 2008, another systematic review by Warnaar N et al. identified 74 cases of intra-operative pulmonary embolism and/or intra-cardiac thrombosis with a mortality rate of 68% (50/74). Twenty recipients were only monitored with a VET device. Eight recipients had signs of hypercoagulability identified by TEG, while in four, the blood samples were clotted before the TEG measurement was initiated. In 2013, Krzanicki et al. reported in their retrospective database review with 124 participants that more than 15% of orthotopic liver transplant recipients were hypercoagulable based on viscoelastic testing. High G-values were present in 15.53% of native TEG, and short Rtimes were reported in 6.80% of native-heparinase TEG. The authors noted that patients with cholestatic biliary diseases had particularly high rates of hypercoagulability (42.9% with primary biliary cirrhosis and 85.7% with primary sclerosing cholangitis). They concluded that SCTs cannot diagnose this condition, and recipients may come to harm if hypercoagulability is unrecognized [8].

In 2014, Clevenger and Mallett discussed the fact that hepatic patients can experience a rebalance of hemostasis. Not recognizing this rebalance can put the patients at risk of hypercoagulability and thrombosis. ROTEM/TEG-guided patient blood management (PBM) protocols aim to avoid inappropriate blood transfusion and coagulopathic bleeding during and after liver transplantation and improve patient outcomes [55]. 

In 2018, Kamel et al. demonstrated in a prospective study on liver recipients a significant postoperative stepwise increase in the FIBTEM (MCF), with a maximum on day 7. Interestingly, this increase in FIBTEM MCF was not associated with elevated fibrinogen blood levels but with an increased incidence of thromboembolic events, as shown in Figure 3 [7].

### 5.4. When to Expect Hypercoagulability in Liver Transplantation?

Hypercoagulability and thrombus formation can happen at any phase of the transplant surgical procedure or after. Wu et al. reported that portal vein thrombosis occurs most frequently in hospitalized liver cirrhotic patients, with an incidence between 0.5% and 16% [56].

Intra-operative: The incidence of intra-cardiac thrombus or pulmonary embolism during liver transplantation ranges from 1% to 6%, as reported by Gologorsky E et al. They witnessed intra-cardiac thrombosis immediately after graft reperfusion in seven patients undergoing liver transplantation [13]. Lerner et al. also reported four similar cases during liver transplantation, even when no antifibrinolytic agent was administered [2]. The intra-operative use of antifibrinolytics as ε-aminocaproic acid (ε-ACA) and tranexamic acid has been advocated as a possible etiology. Accordingly, the use of any antifibrinolytics or coagulation-promoting agents should only be used in the case of bleeding and pathologic TEG/ROTEM results and not on prophylactic bases [3,57,58]. Protin et al. in 2016 also published a case report on acute intra-cardiac thrombosis during the anhepatic phase in a hemodynamically stable recipient [59].

Intra-operative transesophageal echocardiography (TEE) monitoring is a valuable tool for the early diagnosis of intra-cardiac thrombosis formation and is a predictor for any deterioration in cardiac function [9].

Postoperative: Salami et al. observed an incidence of 4.58% of thrombotic events, manifested as vascular thromboembolism in the portal vein or hepatic artery [5]. Arshad et al. pointed to the association between pre- and postoperative thrombosis. This phenomenon is related to the fact that hypercoagulability seen pre-operatively does not resolve immediately after transplantation [51].

### 5.5. Platelet Role in Hypercoagulability

ESLD may suffer from thrombocytopenia due to platelet sequestration in the spleen and hypersplenism [20,21]. Thrombocytopenia seems to balance the increase in platelet aggregation that results from the increase in vWF and the decrease in ADAMTS13 activity [4]. Restrictive platelet transfusion in this condition and during liver transplantation is crucial to avoid exacerbating portal hypertension and increased mortality.

Yassen et al. and Hegazy et al. present two studies in liver transplant recipients that look into platelet functions during surgery and for 21 postoperative days with the ROTEM platelet device. They reported low platelet function and count before and during the transplant procedure. The platelet function took two weeks following transplantation to recover. This recovery was significant among survivors but reduced in non-survivors. TRAPTEM, which is a ROTEM platelet function assay activated by thrombin receptor-activating peptide 6, was able to discriminate 3 month survivors from non-survivors as well as patients at risk of hypercoagulability. In both studies, ROTEM platelet function exceeded the upper normal range in a few recipients on postoperative days 14 and 21. This significant increase in platelet function was associated with thrombocytosis [18,19].

### 5.6. Splenectomy Enhances Hypercoagulability

Intra-operative splenectomy during living donor liver transplantation removes the major site of platelet destruction and reduces antibody production, resulting in prolonged platelet survival times and thrombocytosis. Yassen K et al. and Hegazy E et al. reported that ESLD lowered platelet function and that it took two weeks for the platelets to recover following transplantation. Here, splenectomy enhanced platelet recovery as early as postoperative day 3 and exceeded the normal range in some recipients by postoperative day 14–21, as shown in Figure 4. This warrants monitoring platelet function beyond 3 weeks after liver transplantation and during the follow-up when recipients are least monitored. The antiplatelet effect of acetylsalicylic acid on platelet function was only expressed in assays activated by arachidonic acid receptors (ARATEM), while other assays such as ADPTEM and TRAPTEM were the least affected [19].

### 5.7. Thromboprophylaxis Required Despite Pathologic SCT Results

One of the key messages of this review is the need for venous thromboprophylaxis for recipients with ESLD undergoing liver transplantation during their hospitalization and at any stage of liver transplantation. Notably, the literature indicates that only a small portion of liver patients receive VTE prophylaxis. Prophylaxis includes sequential mechanical muscle compression during surgery and/or pharmacological anticoagulation in the postoperative period. This can range from unfractionated heparin infusion to subcutaneous low-molecular-weight heparin injection and oral aspirin. Pharmacological prophylaxis varies from one transplant center to the other as a result of the lack of an evidence-based unified VTE prophylaxis protocol for liver transplant recipients [60,61,62].

The reason why no guidelines have been established yet may be due to the fear of bleeding. The lack of evidence and the small number of randomized controlled trials in this field puts thromboprophylaxis for liver patients in a low evidence-based category. European guidelines cautiously suggested the need for the judicious use of low-dose unfractionated heparin or LMWH (Grade 2C Recommendation). The low levels of antithrombin due to reduced hepatic synthesis and increased consumption represent a challenge with heparin therapy [57,58,63,64]. Thromboprophylaxis for liver transplant recipients and chronic liver disease patients should consider individual variations and differences between liver diseases. Hospitalized and immobilized patients with cirrhosis without contraindication should receive VTE prophylaxis with either LMWH or UFH. (Level III, Grade C) [33,65,66,67].

**Portal vein thrombosis (PVT) prophylaxis:** This remains controversial and relatively unexplored, with no consensus guidelines [68]. Villa et al. demonstrated that enoxaparin is promising and safe in the prevention of PVT. Furthermore, this low-molecular-weight heparin reduced bacterial translocation and hepatic decompensation, and improved survival [69]. Cirrhotic hepatic patients awaiting liver transplantation or hepatic resection could benefit from PVT prophylaxis as stated by von Köckritz et al. [70].

### 5.8. Hepatic Artery Thrombosis (HAT) Prophylaxis

The prophylactic administration of unfractionated heparin and low-molecular-weight heparin in the immediate perioperative period, to reduce the incidence of HAT among recipients with a high risk of developing HAT, should be considered on an individual basis. Recommendations for or against aspirin to prevent HAT are weak. (Level of evidence III, grade C) [71,72].

### 5.9. Therapeutic Options for Vascular Thrombus

Vascular thrombosis represents an emergency condition that requires immediate clinical intervention to restore adequate liver graft perfusion during or after transplantation. The management varies from one transplant center to the other and is subjected to variations according to the clinical status of each recipient. Anticoagulants represent the primary tool available for most physicians to resolve most vascular thrombosis. Anticoagulants enhance the recanalization of main vessels as the portal vein by dissolving the thrombus. However, alternative anticoagulants, such as the intravenous direct thrombin inhibitor argatroban, need judicious administration in patients with liver dysfunction since it is mainly eliminated by the liver [73,74]. There is no anticoagulant of choice; low-molecular-weight heparin (LMWH), unfractionated heparin (UFH), vitamin K antagonists (VKAs), and direct oral anticoagulants (DOACs), such as apixaban, rivaroxaban and dabigatran are considered on an individual basis (level III, grade C).

**Portal vein thrombosis (PVT) management:** This varies from one recipient to another. In general, the treatment consists of anticoagulation for patients with Child-Turcotte-Pugh (CTP) score Class A or B cirrhosis; CTP is a score used to assess the severity of cirrhosis. VKAs, LMWH, or DOACs require haematology consultation, particularly in advanced liver disease of CPT Class B or **C** scores. (Level III, Grade C). The interventional radiology insertion of a transjugular portocaval shunt (TIPS) is an effective method for the treatment of chronic PVT and portal hypertension (Level II, Grade B) [33].

**Hepatic artery thrombosis (HAT):** HAT is diagnosed by Doppler ultrasonography and requires immediate thrombectomy via a surgical or an interventional radiology approach [33,75].

**Acute intra-cardiac thrombus (ICT): ICT** is not necessarily associated with hemodynamic instability nor does it always require treatment [59]. Routine TEE monitoring during transplant surgery is encouraged as it helps in the early recognition of ICT formation, before the development of hemodynamic instability. Heparin is preferred for recipients with ICT/PE and without hemodynamic instability. Recombinant tissue plasminogen activator (rTPA) (0.5 to 4 mg) is considered for recipients with hemodynamic stability and the dose depends on the degree of the instability (level IV, grade D) [33].

### 5.10. Can VET Monitoring Reduce Thromboembolic Events?

De Pietri et al. [76] discussed how hemostasis rebalance among liver patients can easily turn towards hypercoagulability because of the enhanced generation of thrombin, increased factor VIII and vWF activity, increased fibrinogen levels, and the hyperactivity of platelets. The close monitoring of this hemostasis rebalance by VET testing combined with improved surgical liver dissection recently led to promising results in reducing major bleeding incidents and thromboembolic events with the VET-guided judicious perioperative use of blood products, pro- and anticoagulants, as well as antiplatelet drugs. There is a lack in prospective randomized trials and intervention studies that focus on VET-guided blood transfusion and therapy versus routine perioperative management in liver transplantation. More studies are still needed to provide higher-grade evidence to support the benefit of thromboprophylaxis among liver transplant recipients.

The introduction of VET devices should be encouraged in liver transplant centers to help in the diagnosis and treatment of the complexity of the dynamics of coagulopathy that are associated with liver transplantation and which can shift from hypo to hypercoagulation from one phase to another during the same transplant procedure and perioperative. VET should be performed repeatedly across the pre-operative, intra-operative, and postoperative phases to guide coagulation management effectively and reduce complications. However, no single test can predict thrombotic events entirely; a multimodal approach that involves more than one test should be adopted. We should avoid treating numbers of the VET parameters without correlating them to the current clinical bleeding status during surgery.

In the United States of America, 92% of liver transplantation centers have access to VET with a utilization rate of 60–80% in liver transplantation [77].

### 5.11. Limitations of VET Devices

VET may not always be faster to yield results compared to SCT, since a few ROTEM parameters such as MCF or ML can take up to 30–60 min to be completed, which is close to SCT. ROTEM tests such as INTEM, EXTEM, HEPTEM, APTEM, and FIBTEM include activators like tissue factor and collagenic acid, which artificially activate the coagulation process. These activators can mask the presence of a hypocoagulable state due to platelet dysfunction. Despite these limitations, VET remains superior to SCT, which only provides an ex vivo quantitative assessment of the isolated parts of the coagulation cascade without capturing the full complexity of the in vivo clot formation process. Native or NATEM, which does not undergo citration, recalcification, or an addition of activators, is rarely performed in clinical settings despite its strong clinical value as demonstrated by Kang et al. [78]. Few consider that native blood with no anticoagulants added is ideal for thromboelastographic studies as it closely resembles the patients’ coagulation.

## 6. Conclusions

VETs are more sensitive and help identify recipients at risk for thrombosis. Evidence-based VET guidelines for prophylaxis and treatment of hypercoagulability needs to be developed. A multimodal approach that includes SCTs and VETs will improve diagnosis and management. Thromboprophylaxis should be implemented for hospitalized patients with chronic liver disease, and individual variations and the liver disease aetiology should be taken under consideration. Anesthesiologists taking care of liver transplant recipients must be aware of these differences as they could affect their hemostasis and blood management decisions.

## Figures and Tables

**Table 1:** Studies with viscoelastic hemostatic tests (VETs) findings among liver transplant recipients compared to SCTs.

Study	Type of Study	SCT Overall Findings	VET Overall Findings	Comments
Prah et al. 1995 [9]	Case report	Hypocoagulable	TEG/hypocoagulable EACA	2 patients/pre-anhepatic/non-survivor
De wolf et al. 1995 [10]	Case report	Hypocoagulable	TEG/hypocoagulable	6 patients/intra-cardiac/reperfusion TEE diagnosed
Manji et al. 1998 [11]	Case report	Mild Hypocoagulable	TEG/mild hypercoagulable\aprotonin	1 patient/reperfusion/PA/Carol’s syndrome/survived following surgical embelectomy
O’Connor et al. 2000 [12]	Case report	Hypocoagulable	TEG/hypercoagulable aprotonin in first and ε-aminocaproic acid (EACA) in 2nd	2 patients/RA/survivors 1st rejection redo transplant and transplant aborted. 2nd cryptogenic cirrhosis
Gologorsky et al. 2001 [13]	Case report	3/7 clotted blood sample	TEG 2/7 hypercoagulable/1/7 lysis, 4/7 N/A 5/7 EACA	7 patients/intra-cardiac/3/7 non-survivor/1/7 embeletomy, t PA 1/7, 5/7 no clot removal
Planinsic et al. 2004 [14]	Case report	Hypocoagulable	TEG/hypercoagulable blood sample clotted and CVVH system clotted	1 patient/pre-anhepatic/RA/survivor TEE diagnosed and surgical evacuation
Ramsay et al. 2004 [15]	Case report	Hypocoagulable	TEG/normal	1 patient/Pre-anhepatic/non-survivor/the decision to use antifibrinolytics should depend on TEG data rather than SCTs.
Warnaar N et al. 2008 [1]	Systematic review	N/A 4/22 blood clotted on sampling	TEG 8/22/hypercoagulable	1 patient/non-survivor/pre-anhepatic/non-survivor
Krzanicki et al. 2013 [8]	Retrospective	Hypocoagulable	TEG/hypercoagulable 34/124 (27.4%) high G value.	1/124 patient portal vein thrombosis treated with thrombectomy 6/124 developed HAT—4 HAT required retransplant 2/124 postoperative PE in primary transplant cohort (1.7%). All survived
Piangatelli et al. 2014 [16]	Case report	N/A	ROTEM/hypocoagulable corrected with coagulation factor concentrates	1 patient/pre-operative complete PV thrombosis/hepatitis C/survivor
Kamel, Y et al. 2018 [7]	Prospective observational Cohort	Hypocoagulable	ROTEM/hypercoagulable postoperative	1/50 (2.0%) PVT and 6/50 (12.0%) HAT mainly after critical care discharge and with high FIBTEM MCF in 57% on POD 3 and 86% on POD 7 despite normal fibrinogen plasma. No intra-operative hypercoagulability observed 1-year survival 62%.
Nascimento et al. 2018 [17]	Case report	Hypocoagulable	ROTEM/normal coagulation in pre- and anheaptic, but hypo in neohepatic until corrected	1 patient/pre-operative/partial PV/alcoholic hepatitis/survivor
Yassen et al. 2018 [18]	Prospective e-poster	Hypocoagulable	ROTEM/hypercoagulable	2 patients/partial HAT or PVT detected by Doppler in 2/40 and 1/40 patients, respectively, with no platelet hyperactivity. Both survived
Hegazy E, et al. 2024 [19]	Prospective	Hypocoagulable	ROTEM/hypercoagulable	1/40 patient developed PVT, another 1/40 HAT, both postoperative and with no hypercoagulation. Died POD 20 and 60 from graft failure and sepsis, respectively/hepatitis C/non-survivor

Abbreviations: TEG, thromboelastography; ROTEM, rotational thromboelastometry; RA, right atrium; TEE, transesophageal echocardiography; CVVH, continuous veno-venous hemofiltration; N/A, not applicable; PV, portal vein; HAT, hepatic artery thrombosis; POD, postoperative day.
